# *Journal of Otolaryngology – Head & Neck Surgery* reviewer acknowledgement 2015

**DOI:** 10.1186/s40463-016-0123-9

**Published:** 2016-02-03

**Authors:** Hadi Seikaly, Erin Wright

**Affiliations:** University of Alberta, Alberta, Canada

## Abstract

The Editors of *Journal of Otolaryngology – Head & Neck Surgery* would like to thank all our reviewers who have contributed their time and expertise to the journal in Volume 44 (2015).

**Sumit Agrawal**

Canada

**Khaled Alnoury**

Saudi Arabia

**Jennifer Anderson**

Canada

**Kal Ansari**

Canada

**Margaret Aron**

Canada

**Rick Balys**

Canada

**Sarfaraz Banglawala**

Canada

**Brittany Barber**

Canada

**Alison Beers**

Canada

**Vincent Biron**

Canada

**Brian Blakley**

Canada

**James Bonaparte**

Canada

**Doug Bosch**

Canada

**Maria Brake**

Canada

**Michael Brandt**

Canada

**Tim Brown**

Canada

**Gian-Marco Busato**

Canada

**Neil Chadha**

Canada

**Yvonne Chan**

Canada

**Shamir Chandarana**

Canada

**Justin Chau**

Canada

**Hardeep Chehal**

United States

**Christopher Chin**

Canada

**Matthew Clark**

United Kingdom

**David Cote**

Canada

**Eduard Damrose**

United States

**Sam Daniel**

Canada

**Brock Debenham**

Canada

**Martin Desrosiers**

Canada

**Allan Detsky**

Canada

**Chris Diamond**

Canada

**Joseph Dort**

Canada

**Jaymi Dumper**

Canada

**Peter Dziegielewski**

United States

**Hamdy El-Hakim**

Canada

**Jeremy Freeman**

Canada

**Elaine Fung**

Canada

**Kevin Fung**

Canada

**Richard Gall**

Canada

**Adrian Gooi**

Canada

**Michael Gupta**

Canada

**Jeffrey Harris**

Canada

**Eric Henry**

Canada

**Michael Hier**

Canada

**Kevin Higgins**

Canada

**Jordan Hochman**

Canada

**Paul Hong**

Canada

**Andre Isaac**

Canada

**Adrian James**

Canada

**Arif Janjua**

Canada

**Caroline Jeffery**

Canada

**Liane Johnson**

Canada

**Jodi Jones**

Canada

**Paul Kerr**

Canada

**Shaun Kilty**

Canada

**Rauf Kum**

Turkey

**Morgan Langille**

Canada

**Jane Lea**

Canada

**Robin Leblanc**

Canada

**John Lee**

Canada

**Boyd Lee**

Canada

**Darren Leitao**

Canada

**Vincent Lin**

Canada

**Kristian Macdonald**

Canada

**Fawaz Makki**

Canada

**John Manoukian**

Canada

**Hani Marzouki**

Saudi Arabia

**Emad Massoud**

Canada

**Damir Matic**

Canada

**Laurie Mclean**

Canada

**Eric Meen**

Canada

**Paul Mick**

Canada

**Tamara Mijovic**

Canada

**Alex Mlynarek**

Canada

**Ashkan Monfared**

United States

**Corey Moore**

Canada

**Andreas Mueller**

Canada

**Nagamani Narayana**

United States

**Suresh Nayar**

Canada

**Raymond Ng**

Canada

**Lily Nguyen**

Canada

**Anthony Nichols**

Canada

**Desmond Nunez**

Canada

**Dan O'Connell**

Canada

**Marie-Jo Olivier**

Canada

**Eng Ooi**

Australia

**F. Gigi Osler**

Canada

**Martin Osswald**

Canada

**Susheel Patil**

United States

**Richard Payne**

Canada

**Ronald Pennings**

Netherlands

**Tom Petro**

United States

**Eitan Prisman**

Canada

**Murali Rajaraman**

Canada

**Jamie Rappaport**

Canada

**Keith Richardson**

Canada

**Matthew Rigby**

Canada

**Joan Robinson**

Canada

**Brian Rotenberg**

Canada

**Kathryn Roth**

Canada

**Luke Rudmik**

Canada

**Mark Samaha**

Canada

**Ravi Samy**

United States

**Jim Saunders**

United States

**Julian Savage**

Canada

**Alex Saxby**

Australia

**David Schramm**

Canada

**Muhammad Shakeel**

United Kingdom

**Ehab Shehata**

United States

**Nael Shoman**

Canada

**Praby Singh**

Canada

**Doron Sommer**

Canada

**Leigh Sowerby**

Canada

**Chris Szeto**

Canada

**Mark Taylor**

Canada

**Mark Tewfik**

Canada

**Glynnis Tidball**

Canada

**Jonathan Trites**

Canada

**Jean-Philippe Vaccani**

Canada

**Allan Vescan**

Canada

**Brian Westerberg**

Canada

**John Yoo**

Canada

